# UPR^mt^ regulation and output: a stress response mediated by mitochondrial-nuclear communication

**DOI:** 10.1038/cr.2018.16

**Published:** 2018-02-09

**Authors:** Andrew Melber, Cole M Haynes

**Affiliations:** 1 Department of Molecular, Cell, and Cancer Biology, University of Massachusetts Medical School, Worcester, MA 01605, USA

**Keywords:** mitochondrial UPR, ISR, stress response, proteostasis

## Abstract

The mitochondrial network is not only required for the production of energy, essential cofactors and amino acids, but also serves as a signaling hub for innate immune and apoptotic pathways. Multiple mechanisms have evolved to identify and combat mitochondrial dysfunction to maintain the health of the organism. One such pathway is the mitochondrial unfolded protein response (UPR^mt^), which is regulated by the mitochondrial import efficiency of the transcription factor ATFS-1 in *C. elegans* and potentially orthologous transcription factors in mammals (ATF4, ATF5, CHOP). Upon mitochondrial dysfunction, import of ATFS-1 into mitochondria is reduced, allowing it to be trafficked to the nucleus where it promotes the expression of genes that promote survival and recovery of the mitochondrial network. Here, we discuss recent findings underlying UPR^mt^ signal transduction and how this adaptive transcriptional response may interact with other mitochondrial stress response pathways.

## Introduction

When healthy, the highly dynamic and interconnected mitochondrial network provides the cell with energy in the form of ATP, cofactors such as heme and iron-sulfur clusters, amino acids, as well as nucleotides^1,2,3^. Mitochondria also serve as hubs for many signaling cascades including those regulating apoptosis and innate immunity^4,5,6^. During mitochondrial dysfunction many of these vitally important mitochondrial processes are compromised. With age, a notable increase in mitochondrial dysfunction occurs in otherwise healthy individuals and this decline is exacerbated in age-related neurological and cardiovascular diseases such as Parkinson's Disease and coronary artery disease, respectively^7,8,9^. The underlying causes of mitochondrial dysfunction in these scenarios include an accumulation of damaged mitochondrial genomes (mtDNA) that normally encode 13 essential oxidative phosphorylation (OXPHOS) components required for the function of respiratory complexes I, III and IV, as well as the ATP synthase^10^.

The remainder of the mitochondrial proteome is comprised of nuclear-encoded proteins (∼1 500 in humans) that are synthesized by cytosolic ribosomes and targeted to each compartment within the mitochondrial network and subsequently imported via the TOM (translocase of the outer membrane) and TIM (translocase of the inner membrane) channels^11,12^. Nuclear-encoded proteins are also susceptible to age-associated damage as they can become misfolded and aggregate^13^, which can be exacerbated by locally produced reactive oxygen species (ROS) during OXPHOS-mediated ATP production^14^. Notably, mitochondrial defects are often pleiotropic. For example, OXPHOS or mitochondrial proteostasis perturbations reduce the rate of mitochondrial protein import by reducing the proton gradient or impairing mitochondrial chaperones, both of which must be maintained for efficient import^15^.

## Cellular responses to mitochondrial dysfunction

Organisms have evolved multiple mechanisms to recognize and resolve dysfunction within the mitochondrial network. Collectively, these mechanisms culminate with a response that recovers organelles that are salvageable and degrades organelles that are beyond repair, ultimately yielding a healthier mitochondrial network.

**Figure 1:**
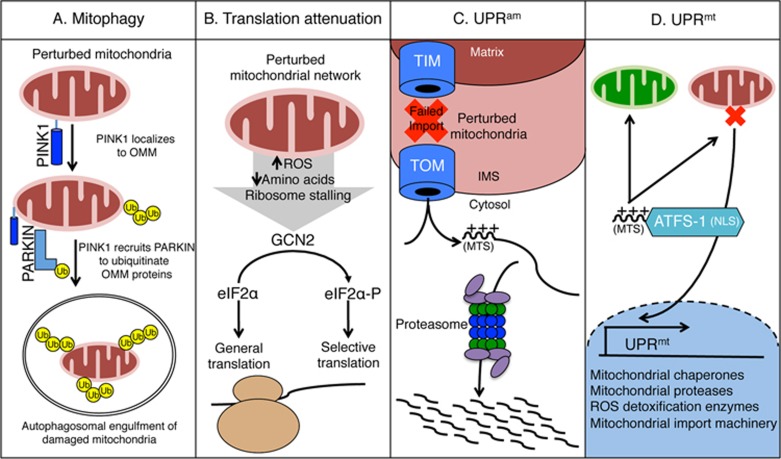
Mitochondrial stress response pathways. **(A)** Recognition and selective degradation of damaged mitochondria is mediated by mitophagy. The kinase PINK1 is stabilized specifically on damaged mitochondria where it recruits the ubiquitin ligase Parkin, which ubiquitinates multiple mitochondrial outer membrane proteins. Ubiquitinated mitochondria are then engulfed by autophagosomes and trafficked to lysosomes where they are degraded. **(B)** The kinase GCN2, which is activated during mitochondrial dysfunction, mediates translation attenuation during mitochondrial dysfunction by phosphorylating the translation initiation factor eIF2α, which serves to reduce the influx of proteins into mitochondria. **(C)** Accumulation of mislocalized mitochondrial proteins in the cytosol stimulates proteasome activity to limit the accumulation of the toxic proteins in a pathway dubbed UPR^am^ (unfolded protein response activated by mistargeted proteins). **(D)** The UPR^mt^ is regulated by the competing organelle targeting sequences in the transcription factor ATFS-1. If ATFS-1 is imported into the mitochondrial matrix via the MTS, the transcription factor is degraded. However, if ATFS-1 cannot be imported due to mitochondrial dysfunction, it is trafficked to the nucleus, via the NLS, to activate transcription.

Severely damaged mitochondria are identified and degraded via the process known as mitophagy (Figure 1A)^16^. Prior to the initiation of mitophagy, severely dysfunctional portions of the mitochondrial network are isolated through fission to prevent stress from diffusing throughout the entire network^17^. Mitophagy requires PINK1, a kinase that is imported into healthy mitochondria and ultimately degraded^18,19^. However, when mitochondrial import is perturbed, PINK1 is stabilized on the outer mitochondrial membrane (OMM) where it phosphorylates ubiquitin^20,21^. PINK1 also phosphorylates the ubiquitin ligase Parkin, recruiting it to the cytosolic face of the OMM where it poly-ubiquitinates multiple proteins^22,23,24^. Poly-ubiquitination serves to recruit machinery that engulfs the damaged organelle into an autophagosome, which is subsequently trafficked to lysosomes for degradation, thus ridding damaged compartments of the mitochondrial network^25,26^.

While mitophagy may represent a last resort for individual organelles, additional stress responses are in place to both limit the damage in defective mitochondria and facilitate the recovery of salvageable organelles. The vast majority of the mitochondrial proteome is synthesized on cytosolic ribosomes and imported into mitochondria, where each protein is processed and assembled with the help of mitochondrial-localized chaperones^12^. If not processed efficiently, the load of imported proteins can perturb mitochondrial proteostasis and impair essential mitochondrial activities. Cytosolic protein synthesis can be modulated during mitochondrial stress by the kinase GCN2, which phosphorylates the eukaryotic initiation factor alpha (eIF2α)^27^, as a branch of the integrated stress response (ISR). GCN2 is likely stimulated by mitochondrial stress via reduced amino acids, increased ROS or ribosome stalling (Figure 1B)^28,29,30^. The phosphorylation of eIF2α results in a decrease in protein synthesis, reducing the number of nascent peptides being imported into the mitochondria^31^.

Reduced protein synthesis may also limit ribosome stalling during co-translational import into mitochondria. Recent work has demonstrated that Vms1, a protein that accumulates on damaged mitochondria by interacting with oxidized sterols^32,33^, resolves stalled ribosomes interacting with the TOM channel^34^. In the absence of Vms1, nascent protein fragments emerging from stalled mitochondrial-localized ribosomes are not accessible to the ubiquitin ligase Listerin due to the tight association with the TOM channel. Upon recruitment to dysfunctional mitochondria, Vms1 prevents the non-canonical addition of C-terminal alanine and threonine (CAT) tails to the nascent peptide as the mitochondrial matrix proteostasis machinery is unable to process CAT-tailed proteins for degradation. In sum, Vms1 localization to the outer membrane of damaged mitochondria prevents the aggregation of nascent protein fragments in mitochondria that can severely impair mitochondrial function.

A consequence of mitochondrial dysfunction is reduced protein import efficiency, which results in the accumulation of mislocalized mitochondrial proteins in the cytosol. A response, known as UPR^am^ (UPR activated by protein mistargeting), promotes the degradation of the highly toxic mislocalized proteins by increasing proteasome activity and reducing protein synthesis (Figure 1C)^35,36^.

The focus of this review is on how cells regulate an adaptive transcriptional response during mitochondrial dysfunction to promote cell survival and recovery of the mitochondrial network known as the mitochondrial unfolded protein response (UPR^mt^). This pathway was initially discovered in mammalian cells, and further characterized in *C. elegans*^37^. The UPR^mt^ is coordinated by multiple factors including the transcription factor ATFS-1 (Figure 1D). ATFS-1 is a transcription factor that promotes the expression of nuclear-encoded genes such as mitochondrial chaperones and proteases, ROS detoxification enzymes, and mitochondrial protein import components^38^. These induced proteins presumably enter functional and dysfunctional mitochondria in the cell to preserve function in healthy organelles and recover activity in damaged compartments. In addition to UPR^mt^ regulation, the functional interactions between UPR^mt^ and translation attenuation, ribosome quality control and the UPR^am^ will be discussed.

## Regulation and transcriptional outputs of the UPR^mt^

UPR^mt^ activation was first described in cultured mammalian cells exposed to ethidium bromide, which perturbs mitochondrial function by depleting mtDNA, resulting in the induction of transcripts encoding mitochondrial chaperones and proteases^39^. Similarly, overexpression of mutant ornithine transcarbamylase, lacking a segment required for proper processing and folding (ΔOTC), in the mitochondrial matrix elicited a similar transcriptional response, indicating a relationship linking mitochondrial function, proteostasis perturbations in mitochondria and UPR^mt^ activation^37^. Subsequent work in *C. elegans* and mammalian systems has identified multiple components required for UPR^mt^ activation, including sensors of mitochondrial dysfunction, regulators of mitochondrial-to-nuclear communication, chromatin regulators, and transcription factors.

### Perturbations that activate the UPR^mt^

Numerous chemical, genetic and proteotoxic stresses have been shown to activate the UPR^mt^, providing clues to the regulatory mechanisms and the physiologic or pathologic scenarios where the pathway may be important^39,40,41^. As mentioned above, the disruption of mitochondrial proteostasis by the expression of a mitochondrial-localized misfolded protein is capable of activating the UPR^mt^^37^. Presumably the misfolded proteins overwhelm the activity of mitochondrial chaperones in the matrix, which is essential for multiple mitochondrial activities including protein import. As such, the depletion of mitochondrial chaperones or proteases is also capable of activating the UPR^mt^^40^.

The impairment of genes involved in diverse aspects of mitochondrial function also activates the UPR^mt^, such as mitochondrial protein import (impairment of *tim-23*), OXPHOS (impairment of complex III or IV), coenzyme Q biogenesis (*clk-1* inhibition), or lipid biogenesis (*acl-12* impairment)^28,40,42,43,44^. In addition, exposure to paraquat, a superoxide generator that perturbs respiratory chain function, causes UPR^mt^ activation^42^ as does the mitochondrial ribosome inhibitor doxycycline^45^. Importantly, all of these perturbations likely reduce mitochondrial import efficiency. By impairing TIM-23, an essential protein import component, transport into the matrix is directly impaired^42^. Respiratory chain perturbations potentially impair import by increasing ROS production and by depleting the proton gradient across the mitochondrial inner membrane. Recently, it has become appreciated that the expression of aggregate-prone proteins in the cytosol, such as mutant Huntingtin protein, linked to the onset of Huntington's disease, also activates the UPR^mt^^46^. How the disruption of proteostasis in the cytosol activates the UPR^mt^ is unclear, however, it is clear that mitochondrial function is disrupted in diseases attributed to aggregate-prone proteins^7,47^.

Numerous studies suggest that UPR^mt^ activation occurs in a variety of human diseases. Mitochondrial disease occurs in 1 of ∼3 000 individuals and assessment of these patients may provide direct insight into UPR^mt^ function^48^. A cohort of patients with mitochondrial disease-associated myopathy (mitochondrial myopathy) correlated positively with an increase in *FGF-21*^49^, a gene known to be upregulated during mitochondrial stress where the UPR^mt^ is activated^50,51^. Additionally, the upregulation of genes indicative of an activated UPR^mt^ has been observed in mouse models of mitochondrial disease^52,53,54^. In addition to mitochondrial diseases, neurodegenerative conditions, including Alzheimer's disease (AD), are associated with mitochondrial dysfunction^7,55^. Recently, it has been shown in AD patient cohorts that an increase in the expression of UPR^mt^-induced genes corresponded with increasing severity of the disease^56^. This includes induced UPR^mt^ genes, such as the mitochondrial chaperone Hspd1 (Hsp60) and the mitochondrial protease Yme1L1 in brain tissue of AD patients^56,57^. These studies in patients and disease models suggest a use for UPR^mt^ genes as biomarkers for mitochondrial disease^49^. Furthermore, the different stressors are informative for our mechanistic understanding of UPR^mt^ activation. Notably, many of the stressors that activate the UPR^mt^ perturb mitochondrial protein import; this commonality between stressors provides mechanistic insight into the activation process.

### Coupling mitochondrial dysfunction to nuclear transcription

**Figure 2:**
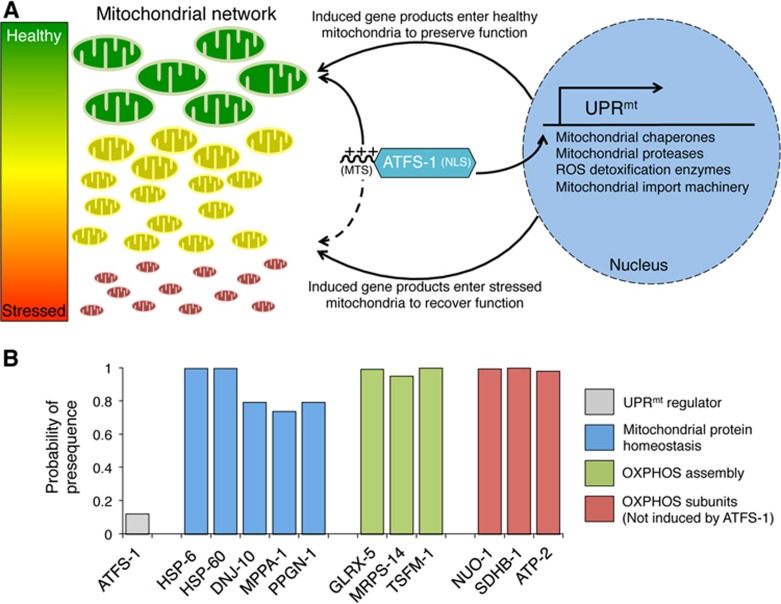
Model by which mitochondrial import efficiency of ATFS-1 and gene products induced by ATFS-1 promote mitochondrial network recovery. **(A)** The transcription factor ATFS-1 harbors both a mitochondrial targeting sequence (MTS) and a nuclear localization sequence (NLS). ATFS-1 is efficiently imported into healthy mitochondria (green), however, import efficiency is reduced by OXPHOS or mitochondrial proteostasis perturbations that cause mitochondrial dysfunction (yellow, red). If ATFS-1 fails to be imported into mitochondria, it is trafficked to the nucleus where it induces transcription of mitochondrial protective genes including mitochondrial chaperones and proteases, antioxidants as well as mitochondrial protein import components. In turn, mitochondrial import of the protective gene products promotes organelle stabilization and recovery. **(B)** Relative to the proteins induced during the UPR^mt^, the program Mitofates^63^ predicts that ATFS-1 has a substantially weaker mitochondrial signal sequence. We hypothesize that a weak MTS allows ATFS-1 to be sensitive to modest mitochondrial dysfunction and translocate to the nucleus. In turn, the strong MTSs in those proteins induced by ATFS-1 can still enter dysfunctional mitochondria with reduced import efficiency to recover function. HSP-6, HSP-60 and DNJ-10 are mitochondrial chaperones, MPPA-1 is a subunit of the mitochondrial presequence processing protease, PPGN-1 is a matrix-localized protease, GLRX-5 is a glutaredoxin that functions in mitochondrial iron-sulfur cluster biogenesis, MRPS-14 is a subunit of the mitochondrial ribosome, TSFM-1 is a mitochondrial translational elongation factor, all of which are induced during mitochondrial dysfunction by ATFS-1. NUO-1, SDHB-1 and ATP-2 are all subunits of the OXPHOS complexes, none of which are activated by the UPR^mt^ (complexes I, II and V, respectively).

In *C. elegans*, the UPR^mt^ is regulated by the basic leucine zipper (bZIP) transcription factor ATFS-1, which contains both a mitochondrial targeting sequence (MTS) and a nuclear localization sequence (NLS)^58^. The presence of dual subcellular localization sequences enables the transcription factor to mediate mitochondrial-to-nuclear communication^42^. Under homeostatic conditions, ATFS-1 is efficiently imported into the mitochondrial matrix and degraded by the protease LON. However, under mitochondrial dysfunction conditions, mitochondrial import of ATFS-1 is reduced, causing it to accumulate in the cytosol. Because ATFS-1 harbors a nuclear localization signal, it then traffics to the nucleus to activate the transcriptional response (Figure 2A). Thus, cells likely utilize mitochondrial import efficiency as an indicator of general mitochondrial function using ATFS-1 as both a sensor and a mitochondria-to-nucleus signaling mechanism. Upon nuclear accumulation, ATFS-1 activates the transcription of over 500 genes that impact diverse cellular activities (Table 1)^38,42,59^.

Once ATFS-1 is imported into the mitochondrial matrix, its MTS is cleaved and the remainder of the protein is degraded, suggesting that mitochondrial import efficiency is a key negative regulator of UPR^mt^ activation^42^. Multiple studies have demonstrated that diverse forms of mitochondrial dysfunction reduce mitochondrial protein import efficiency^18,35,42,60^. The percentage of ATFS-1 that fails to be imported into mitochondria traffics to the nucleus and activates the nuclear transcriptional response^42^. In support of this model, mutations that cause amino acid substitutions within the MTS of ATFS-1 prevent the protein from being imported into the mitochondrial matrix, and result in constitutive UPR^mt^ activation^61^. Thus, UPR^mt^ activation occurs when the import efficiency of the mitochondrial network is reduced.

One aspect of the UPR^mt^ that remains unclear is that if mitochondrial import of ATFS-1 is reduced, how are those gene products induced by ATFS-1 such as mitochondrial chaperones and proteases imported into the dysfunctional mitochondrial network? This issue is partially resolved by the ATFS-1-mediated induction of genes encoding components of the mitochondrial import complexes such as *timm-17* and *timm-23*^42^. In addition, import of UPR^mt^-induced gene products is likely biased towards competent or healthier organelles (Figure 2A). However, this still does not address how or whether the UPR^mt^ recovers dysfunctional mitochondria. One possibility relates to the strength of the MTS on ATFS-1 relative to those proteins induced during the UPR^mt^ such as mitochondrial chaperones and proteases^62^. The program MitoFates analyzes amino acid composition, including net positive charge, to predict the likelihood that a specific amino terminal sequence will be imported into mitochondria^63^. Interestingly, MitoFates suggests that ATFS-1 has a significantly weaker MTS than the mitochondrial-targeted chaperones and proteases induced by ATFS-1 (Figure 2B). This comparison suggests that the relatively weak MTS on ATFS-1 may allow the transcription factor to serve as a sensor of mitochondrial import efficiency. While a percentage of ATFS-1 may fail to be imported into dysfunctional mitochondria, the strong MTSs on mitochondrial chaperones and proteases may permit import into dysfunctional mitochondria to re-establish proteostasis and promote organelle recovery.

### UPR^mt^ activation requires mitochondrial stress-induced chromatin remodeling

The importance of chromatin structure in the regulation of transcription is well established^64^. Interestingly, recent studies demonstrate that chromatin is specifically remodeled during mitochondrial dysfunction to promote UPR^mt^ activation^65,66^. The histone methyltransferase, MET-2 in concert with LIN-65^65^, along with two jumonji domain histone demethylases, JMJD-3.1 and JMJD-1.2^66^, were recently found to be required for UPR^mt^ activation. Both the histone methyltransferase and histone demethylase activities are stimulated by mitochondrial dysfunction. Interestingly, MET-2 and LIN-65 promote global chromatin condensation, whereas the histone demethylases maintain the promoters of UPR^mt^-induced genes in an open or transcriptionally competent state. This chromatin state is further stabilized by the homeobox protein DVE-1 and ubiquitin-like protein UBL-5, both of which are also required for UPR^mt^ activation^65,67,68^. Interestingly, JMJD-3.1 and JMJD-1.2 are both necessary and sufficient for UPR^mt^ activation and stimulated during mitochondrial dysfunction in a manner independent of ATFS-1^66^. Combined, these findings demonstrate the requirement for at least two inputs to mediate UPR^mt^ activation presumably to appropriately match UPR^mt^outputs or strength of activation to related aspects of animal physiology such as development and aging.

### UPR^mt^ regulation via inter-cellular communication

In addition to the cell-autonomous UPR^mt^ regulation discussed in previous sections, UPR^mt^ activation can be communicated between cells and tissues via endocrine signaling. Cell-non-autonomous UPR^mt^ activation has been described using multiple neuronal-specific mitochondrial stressors, which causes intestinal UPR^mt^ activation^43^. The expression of an aggregate-prone mutant Huntingtin protein in neurons is capable of inducing the UPR^mt^ elsewhere in the organism, which requires serotonin secretion^46^. Similarly, disruption of mitochondrial proteostasis specifically in neurons by utilizing CRISPR/Cas9 to impair the protease SPG-7 also resulted in intestinal UPR^mt^ activation^69^. This approach led to the discovery of a second secreted factor, the neuropeptide FLP-2, and a neuronal circuit as being required for cell non-autonomous signaling. Endocrine- or mitokine-regulated activation of the UPR^mt^ likely serves to coordinate activation between tissues, potentially as an early warning system linking sensory neurons that prime a defense for a future mitochondrial stress in distal tissues.

### Mammalian UPR^mt^ regulation

While the initial discovery of the UPR^mt^ was made in cultured mammalian cells^39^, many of the genes required for UPR^mt^ activation were identified in *C. elegans*, owing to the relative ease of using the organism to perform genetic screens^67,70^. Interestingly, numerous recent studies in mammalian systems have suggested considerable conceptual and mechanistic overlap between UPR^mt^ signaling in the two systems although added layers of regulation likely exist in mammals^71,72,73^. For example, a functional ortholog of ATFS-1 was recently discovered. ATF5 is a bZIP transcription factor regulated by mitochondrial import efficiency similarly to ATFS-1. Importantly, ATF5 expression is capable of restoring UPR^mt^ activation when expressed in nematodes lacking ATFS-1. Furthermore, in cultured cells ATF5 promoted OXPHOS and cell growth during mitochondrial dysfunction by inducing expression of several mitochondrial chaperone and protease genes^73^.

**Figure 3:**
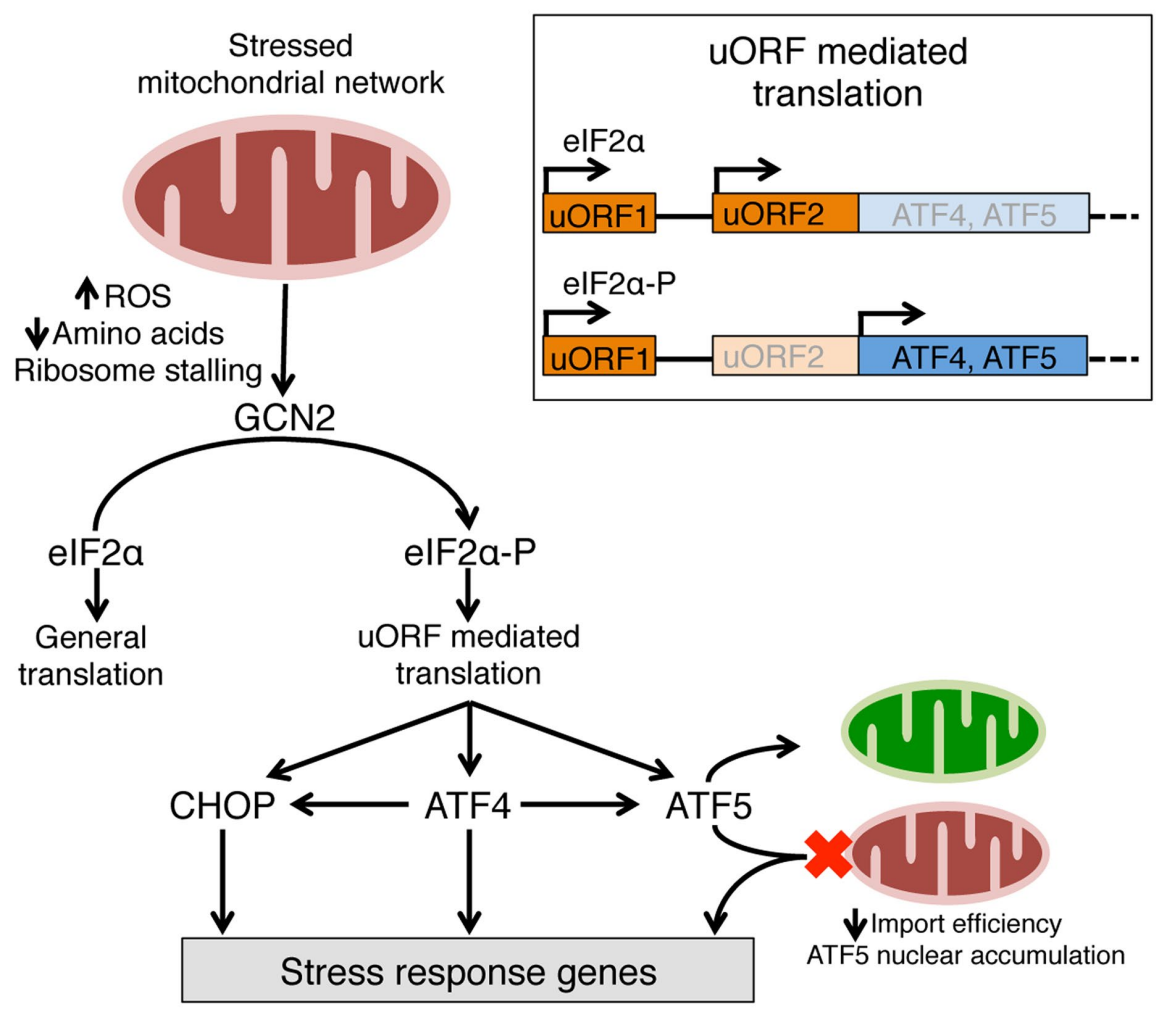
The mammalian UPR^mt^ is intimately associated with the integrated stress response (ISR). During mitochondrial dysfunction, the translation initiation factor eIF2α is phosphorylated by one of four eIF2α-specific kinases such as GCN2 (also PERK, PKR and HRI). eIF2α phosphorylation results in reduced protein synthesis with a concomitant increase in translation of those mRNAs harboring uORFs in the 5′ UTR. The mRNAs encoding the transcription factors CHOP, ATF4 and ATF5 all harbor multiple uORFs and are preferentially translated during mitochondrial dysfunction (inset). While the precise relationship between these three transcription factors remains to be determined during mitochondrial stress, all three are required for the induction of genes associated with the UPR^mt^. Both CHOP and ATF4 induce transcription of *Atf5*. Like ATFS-1 in *C. elegans*, ATF5 harbors a mitochondrial targeting sequence potentially allowing it to specifically respond to mitochondrial stress via reduced mitochondrial protein import efficiency.

In addition to ATF5, at least two other bZIP transcription factors, ATF4 and CHOP, are also involved in UPR^mt^ activation^72,74,75,76,77^. The relationship between ATF4, CHOP and ATF5 during mitochondrial dysfunction remains to be determined. However, it is clear that the expression of all three transcription factors requires the ISR^78,79^. Hence, the activation of the ISR is necessary for UPR^mt^ activation in mammals. ISR activation is mediated by four kinases that phosphorylate eIF2α in response to specific stresses (Figure 3). The ISR kinase PERK responds to unfolded protein accumulation in the endoplasmic reticulum, PKR responds to cytosolic double stranded RNA, and HRI is activated by heme depletion^80^. GCN2 is activated by mitochondrial stress as well as by amino acid depletion, ROS and ribosome stalling^28,81^. Phosphorylation of the translation initiation factor eIF2α results in reduced protein synthesis, but preferential synthesis of mRNAs harboring small upstream open reading frames (uORFs) in the 5′ untranslated region (UTR).

Selective translation mediated by eIF2α phosphorylation requires one or more uORFs upstream of a primary open reading frame (ORF) in the 5′ UTR (Figure 3, inset). Following translation of the first uORF, the ribosome dissociates and the 40S subunit continues to scan the mRNA for the next ORF. In the absence of eIF2α phosphorylation, translation re-initiation occurs quickly resulting in translation of the second uORF, which overlaps the translational start site (methionine) of the primary ORF, preventing translation. However, if eIF2α is phosphorylated, regeneration of the initiation complex is slowed, allowing the ribosome to scan through the start codon of the second uORF, thus enabling the ribosome to engage the primary ORF at a higher rate (Figure 3 inset)^81,82,83^. ATF4, CHOP and ATF5 are three such proteins that require eIF2α phosphorylation to be synthesized due to the presence of uORFs in the 5′ UTR of the mRNA encoding each protein^78,79,84,85^.

Thus, in mammals, UPR^mt^ activation requires eIF2α phosphorylation and is intimately associated with the ISR. However, in nematodes the UPR^mt^ does not require eIF2α kinases (GCN2) or eIF2α phosphorylation; thus, the transcriptional response functions in parallel to the regulation of translation^28^. The translational attenuation likely complements the transcriptional response by reducing the nascent protein load in the matrix, so that UPR^mt^-induced chaperones and proteases may better promote proteostasis in the mitochondria. In mammals, translation attenuation is required for the transcriptional response to mitochondrial dysfunction.

Beyond all requiring eIF2α phosphorylation, the functional relationship between ATF4, CHOP and ATF5 during mitochondrial stress remains unclear. One possibility is that transcription of ATF5 is regulated by both ATF4 and CHOP, which has been shown previously, but not in the context of mitochondrial stress^78,79^. Once expressed, ATF5 can subsequently activate mitochondrial-specific stress response genes if the mitochondrial import of ATF5 is reduced resulting in the nuclear localization of the transcription factor (Figure 3)^73^. However, CHOP and ATF4 likely contribute to transcriptional adaptations to mitochondrial stress directly as well.

Surprisingly, recent work has implicated mTORC1 (mechanistic target of rapamycin complex 1) in UPR^mt^ regulation also via uORF-mediated translation regulation. mTORC1 is a kinase that regulates cellular growth in response to cellular nutrition, ATP depletion as well as growth factors^86^. mTORC1 activation stimulates protein synthesis by phosphorylating S6 kinase^87^, and impaired mTORC1 results in reduced protein synthesis which coincides with increased autophagy^88,89^. Interestingly, the affected tissues in a mouse model of mitochondrial myopathy driven by the accumulation of deleterious mtDNAs, had increased mTORC1 activation and ATF4 activity^54^. While it is unclear how increased mTORC1 activity promotes ATF4 synthesis, it does require uORFs^90^. mTORC1 activation in mitochondrial myopathy also activated ATF5 along with multiple metabolic genes (see below), which were impaired by treatment with rapamycin, demonstrating a requirement for mTORC1 signaling. This work raises a number of exciting questions including how mTORC1 is stimulated during mitochondrial dysfunction and how or whether mTORC1 interfaces with the ISR to regulate the UPR^mt^?

## Metabolic adaptations during mitochondrial stress

As mitochondria play a central role in metabolism by producing ATP, amino acids, lipids and nucleic acids^2,3^, it is perhaps not surprising that the expression of many metabolic genes is altered during mitochondrial dysfunction via the UPR^mt^^42^. For example, ATFS-1 binds the promoters of all glycolysis genes driving their induction during mitochondrial stress presumably to allow the cell to maintain ATP levels, independent of mitochondrial function^38^. Alternatively, ATFS-1 binds the promoters of many TCA cycle and OXPHOS genes, but represses or limits their transcription during mitochondrial stress^38^. Reducing the rate of OXPHOS complex biogenesis while maintaining basal metabolic function through glycolysis may be protective by (1) restricting the amount of ROS byproducts, (2) reducing the load of unassembled OXPHOS components in mitochondria where proteostasis is perturbed, and (3) to reestablish the stoichiometric balance of mitochondrial- and nuclear-encoded OXPHOS subunits.

Metabolic adaption to mitochondrial stress has also been observed in mammalian systems. For example, OXPHOS-deficient cells isolated from mitochondrial disease patients have been shown to survive in culture by increasing glycolysis^91^. In addition, mammalian models of mitochondrial stress have shown that ATF4 promotes one-carbon metabolism^72,92^. One-carbon metabolism is a general metabolic process providing single-carbon units for an array of biosynthetic processes including nucleotide and amino acid biosynthesis^93^. Furthermore, mTORC1, noted above for its role in UPR^mt^ activation, promotes purine synthesis^90^. While there is an immediate benefit for maintaining cellular function through metabolic alterations, the long-term implications of a constitutively active UPR^mt^ that promotes glycolysis and biosynthetic processes may be detrimental as this shift is characteristic of highly proliferative cells.

## Physiologic roles of mitochondrial stress response

### Mitochondrial stress responses during aging

A decline in mitochondrial function with age has been well documented in model systems including yeast, worms, flies and mice as well as in tissues isolated from patients^7,94,95,96,97^. The overall decline in mitochondrial function is highlighted by reduced oxygen consumption with a corresponding reduction in respiratory complex activity^94,95^. Notably this decline has been attributed to the onset of many age-related physiological disorders such as Parkinson's disease and coronary artery disease^7,8,9^.

One of the first described physiologic roles of the UPR^mt^ was during the lifespan extension caused by modest perturbations in OXPHOS. Mutations that perturb OXPHOS or coenzyme Q biogenesis in nematodes, flies and mice have increased longevity^98,99,100,101,102^. The mitochondrial perturbations that lead to the extension of lifespan also cause activation of the UPR^mt^^43^. Importantly, the development and increased longevity of these animals requires multiple UPR^mt^ components, such as *jmjd-3.1*, *haf-1* and *dve-1*, demonstrating that the pathway is protective during mitochondrial dysfunction^65,66,100,103,104^. Furthermore, UPR^mt^ activation by neuronal overexpression of the Jumonji histone demethylase is sufficient to extend worm lifespan^66^. However, which UPR^mt^ outputs contribute to development and longevity remains unclear.

Along with UPR^mt^ activation^43^, mitophagy has also been shown to decline with age^105^. Impressively similar to increased UPR^mt^ activation, increased mitophagy, which is regulated by PINK1 and Parkin, as well as the bZIP transcription factor SKN-1 (Nrf2 in mammals), also prolongs worm lifespan^106,107,108^. SKN-1 is induced by ATFS-1 during mitochondrial stress as are other mitophagy components^42^, suggesting some degree of coordination between mitophagy and the UPR^mt^ during aging, but the precise relationship remains to be determined.

### The UPR^mt^ in aging stem cells

The role of the UPR^mt^ in longevity has primarily been examined in *C. elegans*, an organism that lacks somatic stem cells. Thus, unlike mammals, worms are unable to regenerate or replace cells in somatic tissues. Importantly, the ability to regenerate cell types declines during aging due to reduced stem cell function^109,110,111^. Maintenance of the stem cell pool relies on a balance between self-renewal, periods of quiescence, and differentiation^112,113,114^. Mitochondria are relatively inactive in quiescent stem cells, which primarily rely on glycolysis for ATP production. However, upon differentiation mitochondrial biogenesis occurs, which is associated with an increase in OXPHOS^115,116^. Numerous reports have linked mitochondrial dysfunction with the decline in stem cell function during aging^116,117,118^. For example, transgenic mice that accumulate mtDNA mutations due to the expression of an mtDNA polymerase lacking proofreading activity age prematurely and exhibit stem cell dysfunction^117,118,119,120,121^.

Recent work has suggested a role of the UPR^mt^ and the sirtuin SIRT7 in maintaining hematopoietic stem cell (HSC) function during aging^122^. During mitochondrial stress, SIRT7 is transcriptionally induced and binds to the transcription factor nuclear respiratory factor 1 (NRF1), which induces transcripts required for mitochondria biogenesis including mitochondrial ribosome components. Importantly, SIRT7 represses NRF1's transcription activity. Thus, HSCs lacking SIRT7 have a higher degree of mitochondrial biogenesis and mitochondrial stress, consistent with increased mitochondrial chaperone and protease transcription. Combined, this study suggests that SIRT7 serves to re-establish mitochondrial proteostasis by reducing the load of newly synthesized mitochondrial proteins. Interestingly, SIRT7-deficient HSCs are prone to aberrant differentiation. Thus, SIRT7 represses mitochondrial biogenesis and promotes quiescence to maintain the HSC pool. As the levels of SIRT7 decrease in aged HSCs, the deregulation of the UPR^mt^ may contribute to HSC dysfunction during aging^122^. It remains to be determined whether ATF4, ATF5 or CHOP regulates induction of SIRT7.

### Mitochondrial stress responses contribute to deleterious mtDNA propagation

The mitochondrial genome (mtDNA) encodes 13 essential OXPHOS components, 2 ribosomal RNAs, and 22 tRNAs required for mRNA translation in the mitochondrial matrix^10^. Most metazoan cells harbor hundreds of copies of mtDNA with 5-10 per organelle^123,124^. Steady-state levels of mtDNA are maintained by mitochondrial biogenesis which includes mtDNA replication, requiring the mitochondrial DNA polymerase and TFAM, a protein that packages mtDNA into nucleoids^125^. In addition, mitophagy affects steady-state mtDNA levels by degrading severely damaged mitochondria^125^.

Given the high number of mtDNAs per cell, a low percentage of deleterious mtDNAs (ΔmtDNA; mutations or large deletions) is well tolerated, presumably due to the high percentage of wild-type mtDNAs^126^. In fact, deep sequencing studies have shown that ΔmtDNAs are found in most individuals^127,128^. However, as cells and organisms age, an individual ΔmtDNA can accumulate to a point that perturbs OXPHOS, which impairs cellular function^127^. The accumulation of ΔmtDNAs likely contributes to the reduction in mitochondrial function that occurs in aging cells such as neurons or muscles, as well as in cancer cells^129,130^. The underlying mechanisms that impact ΔmtDNA dynamics remain unclear, but recent work suggests antagonistic roles for the UPR^mt^ and mitophagy.

Perhaps not surprisingly, in a *C. elegans* model of deleterious heteroplasmy (cells containing both wild-type mtDNA and ΔmtDNA) the UPR^mt^ is activated, as the ΔmtDNA lacks the genes required for the expression of several OXPHOS subunits^59,131^. In addition to reduced OXPHOS activity, the heteroplasmic worms displayed mitonuclear protein imbalance as the OXPHOS complex-encoding mRNAs expressed from both wild-type mtDNAs and ΔmtDNAs were overexpressed relative to nuclear-encoded subunits, suggesting additional proteostasis perturbations^59,103,131^. Presumably, UPR^mt^ activation occurs in an attempt to maintain proteostasis and promote recovery of mitochondrial dysfunction, similar to what occurs when the mitochondrial dysfunction is caused by a toxin or a mutation within a nuclear-encoded OXPHOS subunit. Surprisingly, deletion of ATFS-1 resulted in a preferential reduction in ΔmtDNA, with a concomitant increase in wild-type mtDNAs. Furthermore, constitutive activation of the UPR^mt^ in the heteroplasmic worm was sufficient to cause increased mitochondrial biogenesis, which resulted in a preferential increase of ΔmtDNAs relative to wild-type mtDNAs^59,131^. Combined, these data demonstrate that a potential consequence of UPR^mt^ activation is the propagation of ΔmtDNAs, however, it remains unclear how the ΔmtDNA outcompetes wild-type mtDNA.

In contrast to the UPR^mt^, multiple studies have demonstrated a role for mitophagy in limiting the accumulation of ΔmtDNAs^59,131,132,133^. Presumably, the organelles in which the ΔmtDNA/mtDNA ratio severely impairs OXPHOS are recognized by PINK1 and degraded via mitophagy. However, those organelles that contain a low percentage of ΔmtDNAs and are able to maintain OXPHOS avoid mitophagy. One possible mechanism by which the UPR^mt^ maintains ΔmtDNAs is simply by maintaining mitochondrial proteostasis and function, thus limiting the detection and degradation of mitochondria harboring ΔmtDNAs by mitophagy. Consistent with this model, when ATFS-1 is impaired in the absence of Parkin, ΔmtDNAs are depleted but not to the levels observed when mitophagy is intact^59,131^.

Conceptually similar results were recently demonstrated in a mouse model of mitochondrial myopathy caused by expression of a mutant version of the mtDNA replicative helicase Twinkle, which causes aberrant accumulation of ΔmtDNAs^54,134^. The mice accumulate a variety of ΔmtDNAs in post-mitotic cells, leading to muscle OXPHOS deficiency. As discussed above, it was demonstrated that the UPR^mt^ was induced in these mice in an mTORC1-dependent manner. Impressively, mTORC1 inhibition resulted in reduced UPR^mt^ activation and limited the accumulation of ΔmtDNAs, which reduced the progression of mitochondrial myopathy. These data also suggest that ΔmtDNA propagation or accumulation is associated with mitochondrial biogenesis^54^. Interestingly, in addition to limiting mitochondrial biogenesis, inhibition of mTORC1 also activates mitophagy at a higher rate in cells containing heteroplasmic ΔmtDNA^135^, suggesting a second mechanism by which the UPR^mt^ may antagonize mitophagy and promote the accumulation of ΔmtDNAs.

## Perspective

In this review, we have focused on the role of the UPR^mt^ in maintaining the mitochondrial network in model systems as well as in disease and disease models. While it is clear that multiple pathways respond to mitochondrial dysfunction and contribute to mitochondrial network homeostasis, data suggesting interactions between the UPR^mt^, mitophagy, translation modulation and proteasome function are only beginning to emerge. For example, these responses are activated in response to similar forms of mitochondrial stress such as ΔmtDNA heteroplasmy or exposure to toxins such as paraquat^131,136^. Furthermore, numerous reports demonstrate increased eIF2α phosphorylation and ISR activation during mitochondrial dysfunction, which not only reduces protein synthesis, but is also required for the preferential synthesis of ATF4, CHOP and ATF5 that promote recovery of mitochondrial network function by adapting transcription^72,78,79,84^. These findings make it clear that the UPR^mt^ in mammals is included within the relatively broad ISR requiring eIF2α phosphorylation. However, the mechanism by which the ISR is specified to respond specifically to mitochondrial stress remains unclear.

Mitochondrial protein import efficiency is a central component common to the regulation of mitophagy, the UPR^mt^ and proteasome stimulation. Impaired import causes ATFS-1 and ATF5 to traffic to the nucleus to activate the UPR^mt^, and PINK1 to accumulate in the mitochondrial outer membrane to initiate mitophagy, and causes the accumulation of mislocalized mitochondrial proteins in the cytosol that stimulates proteasome activity to maintain cytosolic proteostasis. While OXPHOS and mitochondrial proteostasis are often perturbed by conditions that activate these pathways, it is currently unclear whether more direct regulation of mitochondrial import efficiency plays a role in coordinating these pathways. Recent work has demonstrated that TOM complex-mediated import is regulated by casein kinase 2 (CK2) and protein kinases A (PKA) in yeast. However, whether TOM or either kinase is regulated during mitochondrial stress is unknown^137,138,139^.

Considerable data indicate that UPR^mt^ activation provides protection during mitochondrial dysfunction; however, there are also negative consequences of prolonged or dysregulated UPR^mt^ activation that occurs in the context of deleterious heteroplasmy. It is clear that UPR^mt^ activation promotes development and extends lifespan during mild mitochondrial dysfunction, suggesting that approaches to enhance UPR^mt^ activation may be useful therapeutics. However, several recent reports demonstrate that prolonged UPR^mt^ activation can exacerbate mitochondrial dysfunction caused by deleterious mtDNA accumulation^54,59,131^. In the context of deleterious heteroplasmy, approaches to impair or limit UPR^mt^ activation improve mitochondrial function. Recent work in mice demonstrates that UPR^mt^ inhibition can be achieved by treatment with rapamycin, providing optimism for future therapeutic approaches. The authors found that mitochondrial dysfunction in mouse muscle cells caused by mtDNA damage resulted in increased mTORC1 activation that was required for increased UPR^mt^ activation. Importantly, treatment of mice with the mTORC1 inhibitor rapamycin reduced UPR^mt^ activation, slowing mitochondrial myopathy progression caused by the accumulation of deleterious mtDNAs^54^.

Here, we have reviewed the progress made in understanding how cells and organisms evaluate and respond to dysfunction in the mitochondrial network and adapt transcription accordingly. Included in the UPR^mt^ are not only transcripts that promote mitochondrial proteostasis and mitochondrial biogenesis, but also metabolic adaptations that promote survival and network recovery. While a number of conserved regulatory components required to signal the UPR^mt^ and their individual functions have been identified, their precise interactions remain to be determined. Furthermore, “mitochondrial dysfunction” likely encompasses diverse physiological scenarios; how the UPR^mt^ is specified or receives inputs during each scenario remains to be elucidated. Considering the recent pace of UPR^mt^-related discoveries, we are optimistic that the answers to these questions and more will be resolved.

## Figures and Tables

**Table 1:** Genes induced during UPR^mt^ in both *C. elegans* and mammalian models

***C. elegans* regulated genes**			
	**Gene**	**Function**	**References**
**Mitochondrial protein homeostasis**	*dnj-10*	Mitochondrial DnaJ, protein chaperone	^38,42,59^
	*hsp-6*	Mitochondrial Hsp70, protein chaperone	^38,59^
	*ppgn-1*	Paraplegin AAA protease (mitochondrial)	^38,59^
	*ymel-1*	Mitochondrial AAA protease	^38,42^
**Mitochondrial protein import**	*tomm-20*	Translocase of the outer membrane subunit	^38,59^
	*timm-17*	Translocase of the inner membrane subunit	^38,42,59^
**Mitochondrial dynamics**	*drp-1*	Dynamin-related protein, mitochondrial fission	^38,42,59^
	*mff-2*	Mitochondrial fission factor	^38,42,59^
**Innate immunity**	*abf-2*	Antimicrobial peptide	^42,104^
	*lys-2*	Secreted lysosome	^42,104^
**Transcription factors**	*atfs-1*	bZIP transcription factor, UPR^mt^ regulator	^38,42^
	*skn-1*	bZIP transcription factor, Nrf2 ortholog	^38,42^
**Metabolism**	*glna-1*	Glutaminase	^38,59^
	*clk-1*	Coenzyme Q biosynthesis	^38,42,59^
	*ldh-2*	Lactate dehydrogenase	^38,42^
**Mammalian regulated genes**			
	**Gene**	**Function**	**References**
**ATF4-regulated**	ATF5	Transcription factor, UPR^mt^ regulator	^72^
	CHOP	Transcription factor, UPR^mt^ regulator	^71,72^
	FGF21	Fibroblast growth factor, mitokine	^50,51^
	ASNS	Asparagine synthetase	^51^
**ATF5-regulated**	mtHSP70	Mitochondrial chaperone	^73^
	LON	Mitochondrial protease	^73^
	HD-5	Antimicrobial peptide	^73^
